# The International Union against Tuberculosis

**Published:** 1921-07-30

**Authors:** 


### The International Union Against Tuberculosis.

THE second International Conference on Tuberculosis, convened in London on the invitation of the Council of the National Association for the Prevention of Tuberculosis, was opened on Tuesday, July 26, at the Institute of Civil Engineers, Great George Street, Westminster. Thirty-nine countries in the International Union against Tuberculosis were represented by over 100 delegates, including Professor Calmette, of the Pasteur Institute. The Marquis Curzon and Sir Alfred Mond welcomed them on behalf of the British Government, and His Majesty sent the following message: "The encouraging success which is already manifest from this co-ordinated effort in the cause of health leads me confidently to hope further advances will attend the efforts of the present Congress, in whose work, following the example of my dear father, I take so deep an interest." Co-ordination was indeed further advanced on the opening day, when the International Union and the League of Red Cross Societies entered into an agreement to work together in the fight against tuberculosis, at all events for the coming year. United effort will thus have its full opportunity, and there will be one centre for international work, the determined aim of which will be to stamp out the plague of tuberculosis from this and all other countries of the world.

Official and formal business occupied the morning and early afternoon of the first day; later the annual meeting was held of the National Association for the Prevention of Tuberculosis, when Sir Arthur Stanley, Chairman of the Association, made the gratifying announcement that the Prince of Wales, following the precedent of his grandfather (under whose direct auspices the Association was founded) and his father, when each was Prince of Wales, has consented to become President of the Association. The annual meeting was further distinguished by an address given in English by Professor Armand Delille, of Paris, on "The Protection of Children against Tuberculosis, with special reference to the Grancher System." For the benefit of those who may not know the working of this system we may explain that its essence is the removal of the child of a tuberculous parent, or who is a member of a tuberculous family, from the infected surroundings. Dr. Armand Delille explained that, basing his work on the fact that tuberculosis is not an hereditary but a contageous disease, the late Professor Grancher, who started his organisation seventeen years ago, removed the children while still healthy from the home, and sent them to the country to peasant families. There they remain under the supervision of the country doctor, leading a healthy open-air normal child's life, attending school, and finding occupation, with other children, in fields or garden, kitchen or laundry, so long as is necessary for the disappearance of contagion in their own family, an average of three years—*i.e.* till the cure or death of the tuberculous parent. If the remaining parent does not ask for the return of the child it is retained till the completion of school age (thirteen), when it is either returned to the parent or placed in the country in some suitable occupation. The results of this system, so far as prevention goes, show that of 2,300 children sent from Paris into the country there were only seven cases of tuberculosis, with two deaths from meningitis three weeks after the children were placed. In other words, a morbidity rate of 0.3 per cent. and a mortality rate of 0.0 per cent., as against 60 per cent. and 40 per cent. respectively in the case of the children of 150 tuberculous families, who remained at home, statistics of whom Dr. Delille gave to the Congress of Rome in 1912.

The system has been extended from Paris to other large cities. The "Œuvre Grancher" is a private charity, but the French Government makes a large grant to it, as does the City of Paris and other cities. Further development is, however, needed, so that at least 100,000 children in France alone may be dealt with. Sir ROBERT PHILIP, in thanking Dr. Delille for his address, said the system was based on definite observation, and the conclusions were entirely in keeping with experiments made in another part of the world by one whose name was honoured, Dr. Trudeau. The Grancher system thus explained was scientifically sound, which was more than could be said for many other systems: it was economically sound, for it takes the child before the tubercle bacillus has done its deadly work; and its moral soundness was undoubted, for a child brought up in infected surroundings with a miserable environment has not the same chance in life, morally, physically, or spiritually, as the child that is transferred to a simple home in the country. Sir Robert Philip paid a tribute to the large humanity in spirit which animates the men who have done some of the best work in all countries. Conspicuously is this the case in France, and he cited M. Leon Bourgeois, M. Louis Pasteur, M. Grancher, and M. Roux, the present Director of the Pasteur Institute, who is also Director of the "Œuvre Grancher." Sir ARTHUR STANLEY, the Chairman of the National Association for the Prevention of Tuberculosis, also blessed the system, which, it may interest our readers to know, is practised to some extent here. The Hastings and St. Leonards Tuberculosis Association has adopted this method for some years past.

